# Certainty of Medicine

**Published:** 1834

**Authors:** 


### Art. V.—Certainty in Medicine.

*An Essay on the Certainty of Medicine.* By P. J. G. Cabanis. Translated from the French, by R. La Roche, M. D.

BY A CORRESPONDENT.

It must be evident to every competent observer, that the medical profession in many parts of our country, is strikingly deficient in the talent and acquirements which the importance of its duties demands. If the art is but too often the subject of ridicule on account of its inefficiency; if physicians have failed to obtain that respect to which their high functions entitle them; if impostors are patronised to the mortification of the regular practitioner, and lying panaceas *not seldom* preferred to judicious prescriptions—the profession may look to itself as a cause, in a great degree, of such evils. Is it not the case in many instances, that physicians, after *finishing their studies*, as it is termed, and commencing practice, abandon whatever *science* they may have attained, and sink at once into mere empirics? Hear them railing against books, and theories, and speculations, and dwelling forever on the exclusive efficacy of individual experience, and you would be apt to imagine that they had joined hands with impostors, to pull down the useful, though still imperfect, fabric of medical science, the establishment of which has called forth, for more than three thousand years, the exertions of some of the most philanthropic and highly gifted individuals. The misfortune is, that the empiricism of a regularly bred physician, is of the most pernicious sort. Ordinary quacks borrow from each other, or adopt into their system remedies sanctioned by the experience of the people, or inherit from predecessors, medicines whose worth long use is reported to attest; but our extraordinary quack shuts his eyes to the light of others' experience; establishing a routine for himself, he never departs therefrom, or revises it by the aid of others' knowledge, to see whether it may not be improved.

Many physicians, without acting so blameably, never come up to proper ideas of the art of physic. Their conceptions of the subjects of medical investigation are confused, and their views of the field of medical research but limited. Neither are they aware of the peculiar difficulties that encompass their art, nor of the methods they must adopt to overcome them. Now it is evident, that contracted notions of the subjects, and ignorance of the difficulties of any science, lead to but meagre and mistaken efforts for its advancement. Hence the vast utility of those works, which, though not strictly practical in their character, give us comprehensive and clear views of the boundaries of an art or science, and point out the best methods of its cultivation.

The essay of Cabanis, on the certainty of medicine, is a work of this kind. The object of the author appears to have been, to establish the claims of medicine to the rank of a science, vindicating it against the smart sayings of wits, and the grave accusations of philosophers. The art of physic, he says, has its foundation in nature, in the wants and instincts of the animal economy. All mankind are liable to pain, disease and death. The sick man seeks to be cured, pain cries for relief, and death is the universal dread. Medicine informs us of all those agents, accumulated by the experience of the world from its beginning, which are calculated to assuage suffering, restore health, or prolong life. Wits and philosophers, therefore, who ridicule and asperse the healing art, only manifest their folly and ingratitude, unless, indeed, they be exalted above the common ills of humanity. They abuse their only hope of safety.

Cabanis, in using the term certainty, as applied to medicine, distinguishes it from the certainty accompanying a mathematical demonstration. It means nothing more with him than a high degree of probability—a probability of the same sort and degree as those which regulate us in the ordinary business of life. It appears to us, that in his zeal to raise medicine to its proper place among the sciences, the author has claimed more for it than can well be substantiated. If we mistake not, he supposes medical science capable of being carried to the same perfection, or rather, admitting of as much certainty, as the other departments of knowledge dependent upon observation. We beg leave, on this point, to differ with our author. In our inquiries into the nature and phenomena of living bodies, we must be content with a far less degree of probability, than in the sciences which treat of the affections and circumstances of inanimate matter. A general reason that may here be given for this fact is, that the phenomena and principles of living bodies are far more complicated and hidden than those of inanimate matter. A more particular reason, in relation to the art of curing disease, we will give presently.

Cabanis, by way of establishing the certainty of medicine, notices, and answers in detail, the various objections of cavilers against our art. These objections are, 1st. Our ignorance of the secret principles of life. 2d. Our ignorance of the nature and proximate cause of disease. 3d. Our ignorance of the essential nature of curative agents. 4th. The want of unanimity in the opinions and practice of medical men. 5th. The extraordinary abilities of every kind required in medical researches. 6th. The complication of diseases, and their modifications from almost innumerable causes. 7th. The variations of remedies in their action, from circumstances unknown to the physician. All these objections, except the two last, the author has answered satisfactorily. These two, we think, he has failed to remove, not from a want of ingenuity, but from the very nature of the case. Including them under one head, we shall endeavor to show their real force.

The perplexing intricacy, complication, and deceptiousness of medical phenomena, must certainly qualify our opinion concerning the certainty of medicine. And first, as it regards etiology. The physician, in the course of his practice, meets with a disease immediately preceded by a multiplicity of circumstances, all of which may have morbific tendencies. His business is now to determine, whether one, several, or all of them, have been instrumental in the production of the malady before him. He should also ascertain their individual power, and modes of action in a separate state, and what modifications they undergo in combination—how far they may counteract or favor each other. Next, their mode of action on the animal economy is to be inquired into—as to the parts or structures they primarily affect, and the peculiarities of the functional disturbances they occasion. Nor must he neglect to examine what circumstances, whether extrinsic or pertaining to the system itself, prevent, mitigate, or enhance, their morbific impressions. He must, indeed, be dull sighted, who does not at once clearly perceive that all these phenomena are so darkly complicated, so intimately blended, the most acute and patient observation will be puzzled in analysing the complex mass, discriminating between appearances and realities, and tracing out individual facts. Facts in pathology are equally difficult to determine. To this day, the profession is not agreed as to the first link in the chain of morbid actions; or as to the order in which they occur. And who is ignorant of the fallacies attending post mortem appearances? The difficulty of discriminating, at times, between congestive phenomena, and the marks of inflammation; of determining whether the morbid local appearances have preceded or followed the general malady; and in fine, of drawing an unerring and universal line between healthy and morbid postmortem conditions—is only equalled by the dogmatism with which theorists and hasty observers have settled all these questions. The symptoms, or outward manifestations of diseases, are also peculiarly perplexing. It is true, accurate descriptions of most maladies have been abundantly written; but how imperfect, after a lapse of nearly three thousand years of observation, is our diagnosis. The knowledge of the essential and peculiar signs of a disorder, is almost in its infancy; and the continuing variety of opinion on this subject is a sufficient evidence of the difficulty of its investigation. So hard is it to disentangle the complex mass of symptoms, and refer each to its proper source, and so deceptive are the characters of disease, one malady affecting at times nearly all the signs of another—giving rise, by various sympathies, to contradictory indications, or becoming complicated in its course with another affection, which also has its corresponding manifestations—that the physician must either have had extraordinary skill, or prodigious vanity, who has not often exclaimed, who is sufficient for these things?

But it is in therapeutics, or the application of the materia medica to the cure of disease, that the deceptiousness of phenomena is most remarkable. The comparative influence of art and nature on the termination of disease, and the ascertainment of the efficacious remedy, where several medicinal agents have been simultaneously employed, are the points, concerning which we are most likely to be deceived. Ascribing to medicine what nature has performed, and giving exclusive credit to one article for virtues which result from its combination with others, or pertain, it may be, solely to them; are exceedingly common faults in our profession—errors, indeed, which cannot always be avoided. Many other sources of misconception beset therapeutical researches. Age, sex, physiological condition, and peculiar circumstances attending the administration of an article, exert a strongly modifying power over medicinal properties, and yet how often are they forgotten! The remedy, too, may be spurious, or it may have lost its activity. There may have been some error in the time of collecting or mode of preparing it, in the manner of preserving it or method of exhibition. Above all, idiosyncrasy is a never-failing source of fallacy, not only in therapeutics, but in all inquiries where the action of agents upon the animal economy is to be determined. Medicines effective on one individual, may be inefficient on another. A cause of disease on one will fall harmless in another. Nor can these idiosyncrasies often be detected by observation. There are no external signs, no indications whatever, to advertise the mind of their existence. When we prescribe a medicine, it must act in subjection to circumstances we cannot exclude, over which we can exert little control, and whose influence cannot be exactly pre-estimated. For the same reason, the power of a morbific agent to produce disease, cannot be measured. It is complicated, too, with other causes, and is subject to influences we can neither abstract, nor accurately calculate. In physics, on the contrary—for example, chemistry and mechanics—we can exclude from an experiment circumstances that would modify the result, or at least estimate the amount of their influence.

Two other circumstances, which Cabanis has neglected to notice, might be adduced as strong objections against the certainty of medicine. We will introduce them, not indeed as affording sufficient reasons for disbelieving in the efficacy or excellency of our art; but as being necessary towards forming a tolerably correct idea of the nature of medical evidence.

The first circumstance is, the imperfection of language, arising from the intrinsic nature of the subjects of medical science. Without deriving our illustration from physiology, or from the theory of disease, in which the use of hard words, without meaning, seems legalised, and where the imperfect language is not unsuited to imperfect ideas—we may refer to the description of the signs of disease, and to its nomenclature. He must, indeed, be a novice, who would think of learning the manifestations of a malady by description, or its nature by nomenclature. The attempt to divide, and subdivide, to generalize and specify, disorders, is justly discarded by many of the most enlightened of the profession. Our knowledge of the distinctive characteristics of different diseases is as yet too meagre, and the line of demarcation between them too finely drawn, (they being strangely blended and complicated with each other) to allow, at this stage of the science, an adequate and a correct nomenclature. As regards the description of disease, it is certainly all right, though not sufficient. For after learning all we can from books, the information is almost useless without eye-sight. It may, indeed, *aid* us in recognising a malady, when for the first time we meet it in practice. But how different are our ideas then, from those we have derived from the mere description. The peculiar, striking traits, or diversities of aspect, presented by different disorders, can no more be depicted in words, than can the characteristic expression or cast of countenance of an absent person. This is why the student is directed to the wards of the hospital, or chamber of sickness; and this, too, explains why the young practitioner is so often bewildered, utterly confounded, at actually encountering, for the first time, a malady, with which in books he had been long familiar.

The second circumstance is, that the nature of medical phenomena scarcely ever admits of experiment. "Experiment," says Professor Leslie, "consists in a kind of trial, or artificial selection and combination of circumstances, for the purpose of searching after remote results." From the terms of the definition, it is easy to see the inapplicability of experiment to inquiries in medicine. In physics, chemistry for example, we do not have to wait for the spontaneous recurrence of a phenomenon, to determine its nature or cause. Experiment enables us to anticipate the results of mere observation.

Guided by certain analogies, or perhaps by no analogies at all, we continue numerous and diverse combinations of circumstances at pleasure, until at length we create the hoped-for phenomenon. And now able to reproduce it when we please, and under various modifications or varieties of circumstances, we at last determine its cause. We cannot so proceed in medicine, men may give themselves to Satan for money, but scarcely their living bodies to the doctor, to advance medical science. We cannot persuade people to submit to the action of various combinations of morbific causes, for the sake of a more accurate etiology; we cannot reproduce a disease at pleasure, that we may more correctly determine its symptoms; we cannot at the first onset of a malady, dissect the live body, that we may detect the structures that are affected and ascertain whether certain inflammations really precede or follow the general morbid action. In remedies, the physician does indeed sometimes experiment, but it is only when he knows not what else to do—not then for science's sake, but for his own.

The materia medica, to a certain extent, is susceptible of experiment. We may try upon ourselves or upon animals, the action of a new article; but such experiments, however numerous, yield but probable inferences. The influence of the article upon a dog may differ vastly from that it will exert on a man—its action upon my system is no exact standard for estimating its action on another. Nor are its effects upon the animal economy in a state of health, sure data for determining its effects upon the same in a state of disease. That the experiment may be satisfactory, the article should be exhibited, as recently or long prepared or collected—in different doses, every form, and diverse combinations; to numerous individuals, of opposite constitutions, and every variety of idiosyncrasy; under the varied circumstances of health and disease; at different seasons and in different climates. All this, it is evident, cannot be compassed by experiment; at least by any other experiment than such as may be made use of by many individuals, at distant periods, and under disimilar circumstances, in the course of their observations.

Analogy too, may furnish, in materia medica, many useful suggestions, but never certain information. Analogies drawn from botanical likenesses, or similarity of chemical composition, may lead to the notion of a resemblance in medical virtues; but the notion must be verified by experience, ere it become a principle of practice.

Our knowledge must therefore be built chiefly upon observation. This, of necessity, renders the art of physic, one of slow progress; and at the same time, our knowledge imperfect and uncertain. An age of close observation may be required to determine that, which experiment, were it in our power, would determine in a day. We might here, for the sake of illustration, adduce various instances exemplifying the tediousness and uncertainty of the method of observation, as necessarily practiced in medical researches—but it is presumable, that no further elucidation of the subject is needed.

It must be recollected, that what we have said, applies only to the doctrine of the cause, nature and cure of disease. Several depatments of knowledge more or less subsidiary to medicine, admit to a greater or less degree, of the use of experiment. Physiology is almost, and chemistry altogether entitled to the name of an experimental science. No improvements in chemistry, however, no additional anatomical or physiological knowledge, and no discoveries in botany will ever, of themselves, greatly advance the art of physic. This, now as ever, we repeat, mainly depends on accurate and long-continued observations of the relations of certain agents to the animal economy.

We had written thus far, when our attention was attracted accidentally by the following remarks of a late writer, on the comparative powers of experiment and pure observation, as methods for the discovery of truth. "By means of experiment," he says, "a kind of practical induction is carried on, almost visibly submitted to the perception of the senses, which promotes and suggests conclusions, as it were, mechanically, and checks the errors to which the abstract operations of the mind are constantly exposed. And in the second place, the great and leading advantage of experiment, is the power which it bestows of creating facts, as it were, at pleasure, by which means the mind may select only those luminous facts, called by Lord Bacon prerogative instances. A few of these, he tells us, speak more plainly than a multitude of common facts, so that a consequence may be deduced indirectly from the comparsion of a few examples, by a simple intuitive act of the judgment.

When pure observation becomes exclusively the purveyor of our knowledge, the case is far otherwsie. Here, we must accept, without choice or selection, whatever nature or accident may please to present. There is no power of varying their condition or of changing their combinations,—no means of decomposing them practically, of adding a circumstance, or of withdrawing a circumstance, and observing the changes which follow."*

The foregoing remarks will perhaps tend to show still more clearly, how much medicine has suffered from the impossibility of practicing experiment, and how uncertain, in consequence thereof, is its evidence.

If our observations be correct, it is evident that Cabanis claims too much for medicine, when he supposes it capable of the same degree of certainty, as the natural sciences generally. But is this any reason why the art of physic should be stigmatized as a useless or pernicious art? Then for the same reason, we may discard ethics, the art of government or political economy as useless. Every day we perform many actions from lower probabilities, than the probability of cure, which physic holds out to the sick man. And if such conduct be reasonable in us, is it not madness in him to reject the proffered aid of medicine? "In questions of difficulty," says Bishop Butler, "or such as are thought so, where more satifactory evidence cannot be had, or is not seen, if the result of examination be, that there appears upon the whole, any the lowest presumption on one side, and more on the other, or a greater presumption on one side, though in the lowest degree greater, this determines the question, even in matters of speculation; and in matters of practice, will lay us under an absolute and formal obligation, in point of prudence and interest, to act upon that presumption, or low probability, though it be so low as to leave the mind in very great doubt which is the truth."† Now medicine may be considered as a collection of probabilities in relation to the causes and remedies of disease, in some cases amounting to what Cabanis calls "*practical* certainty," in others constituting but a low presumption, but altogether entitling the healing art to the respect and confidence of mankind. "Far from being," says our author, "as asser-

* Dr. Tod's Book of Analysis, p. 17.

† The Analogy of Religion, Natural and Revealed, &c., by Joseph Butler, LL. D. &c.

ted by some exaggerated minds, the scourge of humanity, the science of medicine is, on the contrary, to be regarded as its hope, its safeguard; and promises in future, to present its resources, which from day to day will acquire greater extension and more efficacy." Nor is this a rash hope. Our science, despite all difficulties, has been steady, though slow in its growth, and now, under more favorable auspices, bids fair to become a magnificent tree, whose branches shall fill the earth, and of whose healing fruit, mankind, with diseases of whatever name, will taste and live. The numerous facts, that have been accumulating for more than two thousand years, have already begun to yield the rich fruits of important principles. A few great laws have been educed from the chaos of the phenomena, enough to redeem medicine from the charge of being altogether empirical, and giving us ground to hope for still more important and useful generalizations.

"I dare make bold to predict that, together with the true method of observation, the spirit of which should always predominate in it, will soon revive in medicine, and that the science will assume a different aspect. The various fragments of which it is now constituted, will be assembled, in order to frame with them a system, simple and fruitful as are all the laws of nature. After a faithful examination of all the facts, and after they have been verified and compared, they will be linked together, and referred to a small number of principles, fixed or susceptible of little variation. The method of this investigation, of uniting by their analogies, or differences, of deduciug from them general rules, which will, in fact, be but a more precise detail of them; this method, I repeat, will be carried to a higher degree of perfection. The important and difficult art, that of applying these general rules to practice, will be simplified." Enough of this bold prediction has been verified, to save the prophet from the charge of presumptuous enthusiasm; and to give us grounds for believing, that medicine is far from having attained that degree of certainty, of which it is capable. Besides, we are to remember that many of the impediments to the progress of medicine, are not only not necessary, but may readily be removed; being dependent upon the errors of physicians, and not the peculiar nature of the art. By noticing some of these errors, the attention of the profession may be awakened to the importance of their rectification; and at the same time we may further the object of our author, which is the advancement of medical science.

1. We note first a want of exactness in following original, valuable recipes.

This is a very common error in our profession, and was long ago noticed by Lord Bacon as an impediment to medical learning. A reputable author may set forth a certain combination of articles, as particularly beneficial in a disorder; but the student does not hold himself bound to remember the proportions of the ingredients. Give me their names, he cries, and I will combine them according to general principles. It is not the fault of the student alone. The enlightened practitioner, misled by the convenient theory of treating disease according to general principles, is apt frequently to deem it a sort of quackery, to adopt precisely the prescriptions of others, however strong the evidence in their favor. He cannot believe medicinal virtues, to depend quite so largely upon the proportions of a mixture, and, in some cases, he takes the liberty of discarding what he conceives a trifling or inefficient ingredient. Now this is altogether wrong. Does he not, in a manner, insulate himself by such a course—cut himself off from whatever advantages are derivable from the multiplied experience of many—and foolishly confine himself to the narrow circle of his own experience? Who does not know, that notable virtues sometimes result from peculiar combinations, even though one or more of the ingredients may be separately inert—virtues indeed, which cannot be obtained by any mode of uniting the active articles alone. Nor is it rare, to meet with a compound whose efficacy is dependent upon the peculiar *proportions* of the ingredients. Dover's powder furnishes a striking exemplification of both these facts. One of the constituents, the sulphate of potass, is, by itself, comparatively an inert substance, in the proportion it holds to the other ingredients. Reasoning from this fact, a priori, we might readily conclude that the mixture would be as efficacious without as with it,—but experience has shown this to be fallacious. There is no combination we can make of ipecacuanha and opium, so excellently sudorific as Dover's powder. And not only so, but any deviation from the established proportion, is apt to diminish the efficacy of the compound. That the same is true of many other combinations, can scarcely be doubted. When, therefore, a person of sufficient judgment and experience, recommends a prescription as possessing peculiar medicinal qualities, we should forbear to modify it at our will, and then because unsuccessful, declare it of no utility. Nor, judging only from the properties of the ingredients in a separate state, should we pronounce it worthless. It would be wiser to employ it in the exact proportions, advised by its author, in the same doses, with the same precautions, under the same circumstances every way,—and then not once, but several times, until we have determined its true worth. In this manner alone, can we judge righteous judgment, and hereby also, we shall avoid in one way, retarding the progress of medicine; for next to embracing the results of fallacious experience, the neglect or rejection of the fruits of correct experience has injured our art.

2. A kindred error, and one frequently giving birth to the fault just noticed, is a disbelief in specific remedies, and a consequent lack of inquiry for them. This we take to be a serious error, and a great impediment in the way of our professsion. So far as our knowledge now extends, there are few remedies of specific power,—a power, I mean, capable of making peculiar impressions on the animal economy over and above their stimulating or sedative influence. With the exception of calomel, iodine, sulphate of quinine, secale cornutum, and perhaps one or two more, our present materia medica may be divided into mere stimulants and sedatives, acting either generally or upon particular parts. Even the specific power of calomel and iodine have been called in question. (It will be recollected, that we use here the term specific, not as applying to an influence directed upon a particular organ, but to a peculiarity in the mode of action.) Sulphate of quinine, probably, is an article which cannot, without absurdity, be denied the possession of specific properties—properties whose impressions upon the living body cannot be supposed of a merely stimulant or sedative character.

The reason of this peculiarity of our materia medica, is to be sought for in the prevalent doctrines concerning the nature of disease. We have been taught to believe, that every deviation from a state of health, consists essentially either in a diminution, an exaltation or inequilibrium of the vital energies. Hence, the inquiry has been for remedies, that would meet these conditions, subduing, augmenting, or equalizing excitement, according as the case may be; and we have neglected to trace further their operation. Hence, too, the exclusion of specifics. Now we are among those, who disbelieve in the universal truth of these fashionable theories of disease. It is not our business here, nor have we time, to go into an examination of them; but we will briefly state the chief grounds of our disbelief. 1st. There may be exaltation, depression or inequilibrium of the vital powers, without disease.—2nd. Mere differences in the intensities of these conditions, do not appear to us sufficient, to account for the innumerable sorts and varieties of morbid action.—3d. The total inefficiency of stimulant, sedative, and revulsive agents, in many maladies, even when most judiciously administered, can only be explained by supposing different disorders to possess specific, essentially distinct natures.—And 4th. The diverse products, or terminations of disease, point to radical differences in kind as well as degree of morbid action. If this doctrine be true, and if, as is the case, we possess but three or four remedies able to exert a specific action on maladies,—to meet diseased conditions with corresponding antidotal powers—radically to affect and peculiarly to restore alterations in the vital properties,—how exceedingly deficient is our materia medica, how devoid of real strength is our art! Do you not see, that we are compelled to combat disease, almost without arms? Physic, says one, is the art of amusing the patient, while nature cures the disease: If he had said, the art of palliating symptoms, while nature cures the disease, his satire would have been no less true than severe. For certainly with our present remedies, we are scarcely able to do more wittingly than obviate the immediate effects, without reaching the hidden source and nature of disease. But this is our own fault. Influenced by famous theories, we have neglected to search out remedies, not sanctioned by them. If we have no certain, specific medicinal substances, it is not because they do not exist. Heaven has never afflicted mankind with peculiar evils, for which it has not provided appropriate remedies. The example of sulphate of quinine and the few other articles we have, possessing peculiar properties, teaches us to confess the reasonableness of belief in specific agents. Nor is there quackery in the idea, for the knowledge of them is to be gained only by long-continued and accurate observation; not by signs, and spells, and guessing, and the other expedients of imposture. Medicine, we believe, is destined to receive some of its greatest improvements from the search after specific remedies in relation to disease;—and a disbelief in their existence, has been among its chief impediments.

3. A third impediment arises from the absurd speculations of men of genius. Theorists have ever been busy in our profession: what pity that the judiciousness of their efforts has not equalled the ambition that prompted them. As it is, their labors have generally cumbered the science, whose march they hoped to forward. A man of genius, as ordinarily understood, will seldom stoop to the drudgery of collecting and examining facts. His vivacious imagination catches at a few phenomena, and lays them down as a foundation for an unsubstantial fabric of speculative principles. We are all addicted to theorize, and can hardly look upon insulated circumstances, without directly seeking out some general laws which may connect and explain them. A theory, though untrue, is said by some to be beneficial,—acting as a stimulus to inquiry, and serving to give order and perspicuity to what facts may be collected. Theory, we answer, takes so firm a hold on the affections, as to alloy the love of truth and bias inquiry—impelling us in search of circumstances to confirm our hypothesis, and concealing from us or distorting those which oppose it. Buside this general way in which false theories act, they also retard the progress of physic by turning the attention of medical men from facts to words, from the investigation of nature, to the disputes of dogmatists. The distinguished theorists themselves, fascinated by their own chimeras or lusting after the praise bestowed on the leader of a sect, are no longer content to be the interpreters of nature, but would be sole directors of public opinion. Metaphysical subtilties, not tangible truths, are the proofs on which they rest their speculations. Inferior minds, enslaved by their authority, blinded by their ingenuity, perverted by their sophistries, or gratified with an opportunity for discontinuing painful research, build up the mass of error, whose foundations their superiors have laid. How great has been the waste of intellectual energy, in the attempt to resolve the multifarious phenomena of medicine into a few elementary truths, with the illusive hope of giving it the form of a demonstrative science. We have already had occasion to notice the evil influence exerted by the prevalent pathological doctrines. As further exemplifications of the pernicious effects of false speculations, we may point to the one-sided, imperfect investigations, which sprung from the notion of Galen, who derived all medical virtues from four elementary or cardinal qualities, heat, cold, moisture and dryness—from that of Stahl, whose practice, consisting, chiefly in a patient waiting upon nature, was felicitously styled, a meditation upon death—and lastly from the mechanical theories.

4. An impediment, nearly allied to the last, consists in the hasty, inadequate observation, and rash, unwarranted generalization of the profession at large. He observes hastily and inadequately, who does not carefully view a phenomenon on every side, and in all its relations; or who esteems that a fact, which, if inquired into, turns out fallacious. He generalizes rashly, who, from a few circumstances, infers a general principle or law, which he extends to other circumstances, connected with the first only by some faint or fanciful analogies. It is owing to the former of these faults, that our journals are so continually flooded with accounts of new, certain, all-powerful remedies, whose worthlessness, however, further experience soon demonstrates. Unwarranted generalization is the source of the vast number of crude theories of perennial growth in our art. It is easy to understand how these faults have retarded medical science. Incorrect statements of phenomena are so many obstacles to be cleared away from our path; and as to rash generalization, its evils have already been pointed out, when speaking of the influence of false theories. All we have further to remark is, that the error is not confined to men of great name. A physician having but three patients per annum, is not often at a loss for some general theory of disease, or operation of medicine; or without some notable remedy, which is made to perform as much service as the warm water of Dr. Sangrado. No matter whether it be a great or little genius who thus generalises, they equally cling to their darling heories; are equally eager in hunting up facts to give them countenance, and equally loath to receive witness against them. How much has medicine suffered from this species of egotism! The two faults we have just noticed are founded very frequently upon the ignorance or inability of professional men.

5. This leads us to consider a fifth impediment, which is the want of a suitable preparatory education among medical students. We might here take occasion to censure, what has so often been censured, that strange misconception of some parents, which induces them to select the most unpromising of their offspring for our profession. The lad of brilliant genius is destined for the bar; he, of the solid parts, is kept at home to manage the estate; commerce claims the quick, shrewd, and enterprising one; while medicine must be cumbered with the blockhead. This mistake can only spring from the grossest ignorance of what talent our art requires. Such parents would do well to recollect in time, how many lives will, in all probability, be sacrificed by their hopeful progeny, ere he learns that, than which *he* can learn no more, how to avoid doing mischief.

We say, that the preparatory education of medical students, is generally ill adapted to qualify them for the successful prosecution of their art. Our notice of this point shall be brief:—we mean not to say, that a student should have previously accomplished himself in the knowledge of the classics—of Latin, Greek, or other tongues. It is not tongues, but minds, that are needed in our science. Love of truth, a capacity for correct observation, and the power of judging good judgment, are the qualities necessary in physic. The first quality is required to keep the mind free from every undue bias; the second, to enable it to collect a sufficient store of available facts; the third, to guard it against deception, to trace the true analogies of phenomena, and infer general truths. Now whatever studies or pursuits are best calculated to endow the mind with these qualities, should be embraced in the preparatory course of the student. Hence the village rustic, who has habituated himself to the observation of human character, and judgment of human actions; or the farmer's boy, who has learned correctly to observe the varieties of soil, and to infer general truths concerning their relations with different productions; has acquired greater abilities for medical research, than the merely learned youth, who can read fluently, in the original, the Georgics of Virgil or Satires of Horace, but without appreciating the wit of the one, or wisdom of the other. We repeat, it is not knowledge, so much as power and discipline of mind, that the medical student requires, in order to make him an efficient member of his profession. Is there not now, has there not been a lack of such preparatory education? And has not the profession suffered severely from members devoid of the requisite abilities? Now, in order to secure the art against such artists—to save the field of medicine from the cumbersome, profitless tillage of such laborers—what is to be done? Let those whose business it is to examine into the pretensions of candidates for graduation, institute a more rigid, searching ordeal. The want of this, permitting the too easy admission of many, who can add neither glory nor benefit to the profession, constitutes a sixth impediment. For proof of this fact we appeal to the experience of the profession. Has not each of us known many a regular, diplomatized physician, whose claims to the confidence of the community our hearts have denied—who has excited in us wonder—wonder that ever a board of examiners, impartial and enlightened, should have styled him "Doctorem in arte medendi—dignum amplissimis honoribus," &c. &c. It is not our business to assign the cause of this—sufficient is it to know that the laxity of examinations is a radical evil in the medical system of this country; and the example of reform must begin in some high place ere the evil will be remedied. As it regards a man's qualifications for the *practice* of physic, we do not believe they can be ascertained, except by questions propounded upon disease before him. It should be determined in the wards of a hospital, whether the student can judge sufficiently well of the symptoms, complications, indications, and particular remedies of the maladies immediately under his notice.

7. A limited view of the subjects of medical investigation, and a foolish, vain confidence that all is known that may be known, have retarded our art, inasmuch as they withdraw all stimulus to inquiry. The remark is made in special reference to novices—to those who have but just stepped into the profession, with their young, blushing honours thick about them. I have heard some of them declare, they now knew as much as any one could teach them. All the treasures of medical wisdom and knowledge they seemed to fancy their diplomas had conferred. It is precisely a small mind that grows so suddenly all-wise and self-sufficient. An opposite error, but one whose influence is equally hurtful, consists in regarding as unascertainable the objects of medical inquiry; or being paralyzed in resolution by the vast difficulties that beset them. But we will not dwell on these points.

8. Another impediment is scepticism in the efficacy of medicine. Total scepticism is a heartless thing—an enemy to all enthusiasm, a drawback to all investigation. For if there be nothing to be learned, why should I yet seek to learn? It is a baneful error, arising sometimes from impatience of examination—often from a want of skill and ability. A practitioner once said, that every year served to diminish his faith in the efficacy of physic; and the remark was made upon having failed to procure alvine evacuations by large exhibitions of drastic purgatives. He forgot to attribute his failure to the right source—a neglect of blood-letting and other relaxing measures previously; for the case was attended by a strongly inflammatory condition. Whenever, indeed, we hear physicians frequently exclaiming against the efficacy of medicine, we deem it a presumptive argument of their want of skill. Their disappointments, most probably, are errores non artis sed artificum.

9. Another impediment, is the indifference of many to the acquisition of profound medical learning, arising from the conviction that such an endowment will be unappreciated. Mediocrity in physic too often supersedes higher merit. Professional men are apt to be slow in acknowledging superior excellence, and as to the people, he who can best humor their fancies is generally the best physician. Exhibitions of medical skill, from their very nature, are unappreciable by the uninitiated. Hence, old women, witches, and impostors, as is said by Lord Bacon, have ever been the rivals of physicians.

10. Another impediment, and we conclude our list. An inordinate love of gain, benumbing the faculties, or prompting them to just so much exertion as may be necessary for the acquisition of wealth; tempting the practitioner to profit by more patients than he can possibly attend, and occupying all his hours with wishes, and schemes, and efforts for private aggrandizement—is a prevailing, serious impediment to the improvement of our profession. For what gifts can science expect from men solely intent on riches, who at the bed-side of a patient are more busied in anticipating the amount of their fees, than in estimating the peculiarities of the case?

We conclude by observing, that it would be wisdom for us to test fairly the virtues of original, reputable recipes—to put away from amongst us the disbelief in specific remedies—to avert or counteract the influence of absurd theories—to avoid hasty observations, and rash generalization—to suffer the influence of no eminent name to fetter or bias our inquiries—to promote improvement in the preparatory education of students, and reform in the examination of graduates—to take neither disheartening views of the difficulties, nor meagre views of the objects of medical science—to learn to distrust ourselves, rather than our art—to permit not the indifference of the world, or our professional brethren, to relax our efforts after excellence—and finally, to animate our bosoms with a fervent, disinterested desire for the attainable perfection of our art.

